# Locked down with my eating disorder: a retrospective study on the impact of COVID-19 lockdown on adolescents with eating disorders

**DOI:** 10.1186/s40337-023-00792-1

**Published:** 2023-05-10

**Authors:** Bianca Borsarini, Edoardo Pappaianni, Nadia Micali

**Affiliations:** 1 Department of Psychiatry, Faculty of Medicine, University of Geneva, Geneva, Switzerland; 2 Center for Eating and feeding Disorders Research (CEDaR), Psychiatric Centre Ballerup, Mental Health Services in the Capital Region of Denmark, Maglevænget 32, 2750 Ballerup, Denmark; 3 Great Ormond Street Institute of Child Health, University College London, London, UK

**Keywords:** Eating disorders, Adolescents, Children, COVID-19, Lockdown, Pandemic, Anorexia nervosa, Bulimia nervosa, Binge eating disorder

## Abstract

**Background:**

Negative effects of COVID-19 lockdowns have been reported in adult patients with feeding and eating disorders (FED) whereas evidence of its impact on young clinical populations is still limited and somewhat inconsistent. The present study aims to investigate the effect of the first COVID-19 lockdown on a range of FED symptoms in children and adolescents: (a) already receiving treatment in our specialist service for FED when the pandemic hit, and (b) prospectively evaluated in our service from October 2020 to July 2021.

**Methods:**

Out of sixty-one eligible patients with a broad spectrum of FED invited, forty-five young patients (aged 11–18) consented to participate and were included. An ad-hoc survey, consisting of open questions, multiple choice questions, yes/no questions, and a symptoms checklist, was administered online.

**Results:**

About half of the participants (46.7%) reported a positive effect of lockdown on FED symptomatology. Patients with anorexia nervosa (AN) reported the highest rate of symptomatology worsening (58.6%). Younger patients (11–13 years) showed a greater improvement of symptoms compared to older ones (14–18 years of age). COVID-19 lockdown was identified as the precipitating factor for FED onset in 60.7% of newly evaluated patients.

**Conclusions:**

Evidence from our investigation points out that although the COVID-19 pandemic was a precipitating factor for a FED for many active and newly referred patients, it had a positive impact on youth who were already in treatment and younger participants.

**Supplementary Information:**

The online version contains supplementary material available at 10.1186/s40337-023-00792-1.

## Plain summary

Negative effects of COVID-19 lockdowns have been reported in adults with Feeding and Eating Disorders (FED) whereas evidence of its impact on young clinical populations is still limited. This study investigates the effect of COVID-19 lockdown on FED symptoms in a clinical population of children and adolescents. Patients were contacted from October 2020 to July 2021 and a survey was administered online. About half of the participants (46.7%) reported a positive effect of lockdown, with younger patients showing greater improvements. Patients with Anorexia Nervosa (AN) reported the highest rate of symptomatology worsening (58.6%). Although the lockdown was the precipitating factor for FED onset in many active and newly referred patients (60.7%), it had a positive impact on those who were already in treatment and on younger participants.

## Background

In March 2020, the COVID-19 pandemic broke out as a unique event with global implications that continue to be a major concern [1, 14]. Extreme safety measures were implemented, such as: national lockdowns, school closures, obligation to work from home, social distancing, compulsory protective masks, quarantine, and isolation. The consequences of COVID-19 lockdowns and closures on mental health (and particularly amongst young people) have been many and have been documented [6, 7, 13, 21].

Negative effects of COVID-19 lockdowns have been reported in adult patients with Feeding and Eating Disorders (FED) [4, 5, 11, 17]. In particular, in the majority of adult individuals with ED, symptomatic deterioration and increased concerns about body shape and exercise have been found [17], as well as increased rumination about disordered eating [5] and co-occurrence of anxiety and depression [17].

Conversely, evidence of effects of the COVID-19 pandemic and related lockdowns on feeding and eating disorders (FED) in youth is limited and findings are somewhat inconsistent [2, 8, 18, 22]. Some studies have highlighted a worsening of FED symptoms following lockdowns [8, 18, 22], on the other hand a positive impact of COVID-19 closures on patients’ motivation to recover and symptom improvement was highlighted [2]. Given these conflicting findings, we aimed to investigate the effect of the first COVID-19 lockdown (Spring 2020) on a range of FED symptoms in children and adolescents: a) already receiving treatment in our specialist service for FED when the pandemic hit, and b) prospectively evaluated in our service from October 2020 to July 2021.

We aimed to:

1. Determine if the first COVID-19 lockdown (March/April 2020) was associated with an improvement or worsening of symptoms, for all patients who were already in treatment and those newly referred;
2. Investigate the associations between individual and family-related factors and changes in symptoms;
3. Quantify the number of newly evaluated cases reporting COVID-19 lockdown and related measures as a precipitating factor for illness onset.
4. Determine satisfaction with treatment during lockdown and access to care.

## Methods and data analysis

All patients, already in active treatment or new cases for a FED in our specialist program AliNEA (Alimentation et Nutrition chez l’Enfant et l’Adolescent), at Geneva University Hospital in Geneva (Switzerland). Our service evaluates and treats children and young people from 4 up to 16 years of age, from the Geneva and neighboring Swiss Cantons with FED. Children and adolescents are referred to us via various routes (self-referral, primary, and secondary care), and are at varying stages of illness (with a range of duration of untreated/treated illness).

Young people and their parents (for those aged 11–18), or parents only (for young people below 11 years of age), who had given consent to be contacted, were eligible to participate.

**Fig. 1:**
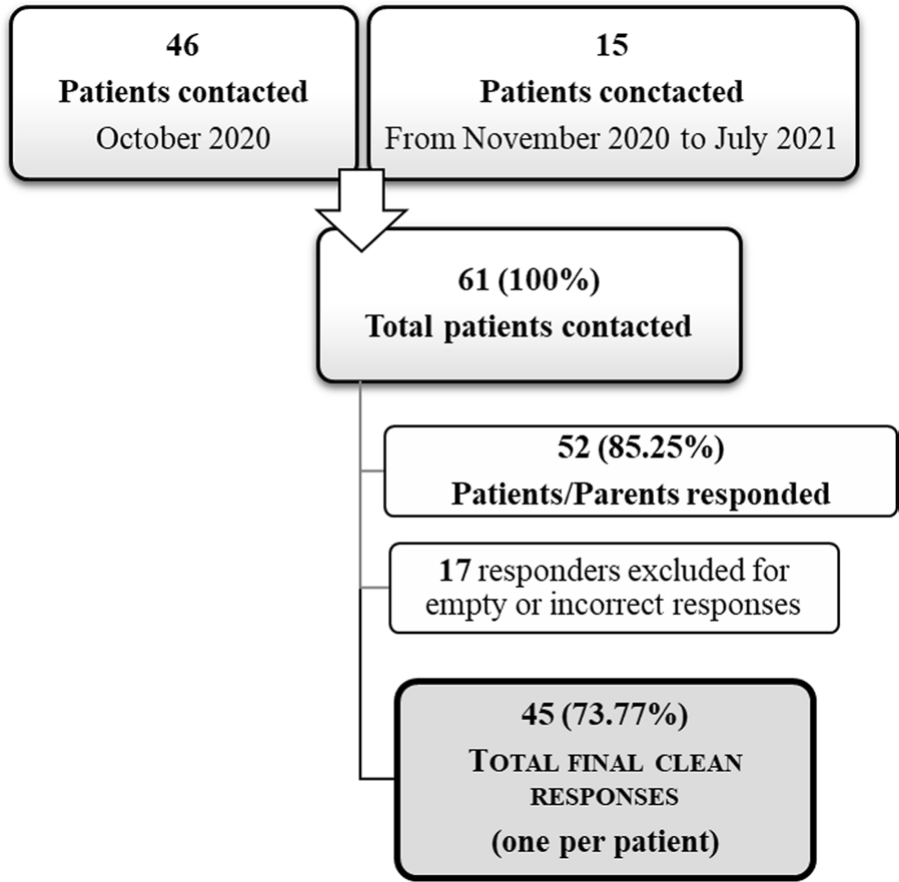
Flowchart describing recruitment timing and response rate

An initial subset of forty-six eligible young patients and/or their parents were contacted by e-mail and letter and were invited to take part in the study in October 2020. Consecutive referrals were contacted between October 2020 and July 2021, as they were referred to the clinic after the initial recruitment round (n = 15 young patients (and parents)) (see Flowchart, Fig. 1).

### Survey

Given that no existing surveys or questionnaires were available to address our research question, we developed an ad-hoc survey in order to bridge this gap.

The survey was administered via REDCap (Research Electronic Data Capture) [9, 10]. It consisted of open questions, multiple choice questions, yes/no questions, and a FED symptoms checklist. The following introductive paragraph opened the survey: “The lockdown due to the COVID pandemic was a difficult and unique period for some. For others, it was a good opportunity to do things differently. We would like to ask you about your experience of lockdown in relation to your (your child’s) eating difficulties.”

Participants were asked:

1. To rate the effect of the first COVID-19 lockdown (March 13th–June 19th 2020) on a range of FED symptoms relative to pre-lockdown on a Likert scale. The symptom list included 12 behaviors and cognitions relevant to FED diagnostic criteria (e.g., food restriction, fear of weight gain, binge eating).

Each response scale ranged from 0 to 100 (0 = much worse than pre- lockdown, 100 = much better than pre-lockdown and 50 = no change in symptoms; 3 anchors were provided);

2. To answer multiple choice questions concerning home arrangements, schooling, meals organization (sharing and preparation);
3. To indicate their worry about their and their family’s health in relation to the pandemic.

Individuals were also asked if they or anyone in their nuclear family had contracted COVID-19. A question about the perceived ease of access to treatment and care by our team during lockdown was included. Socio-demographic, FED diagnosis, and other baseline data were obtained as part of routine clinical collection at first clinical assessment.

### Clinical and related variables

FED diagnoses were made by the multidisciplinary team according to DSM-5 criteria (5th ed., DSM-5, [3]), discussed and agreed in multidisciplinary meetings where two senior child and adolescent psychiatrists were present. Treatment duration was obtained from clinical notes and is presented in months.

For prospective cases with FED, we examined whether COVID-19 lockdown was recognized by the family and the youth as a precipitating factor for the illness from clinical notes (precipitating factors are systematically recorded in a structured formulation written at assessment and sent to families).

### Baseline clinical assessments

*Anxiety* State and trait anxiety were assessed using the Spielberger State-Trait Anxiety Inventory (STAI) [19] a 40-item self-completed questionnaire that aims to assess separately state anxiety and trait anxiety via 2 sections of 20 items each. Two versions of the inventory were used depending on the age of the participants: the STAIC (State-Trait Anxiety Inventory for Children, [20]) for children < the age of 14 years and the STAI-Y [19] for those ≥ 14 years (Internal consistency: from 0.86 to 0.95; test–retest reliability: from 0.65 to 0.75 [19])).

*Family functioning* Family features were assessed using the Family Adaptability and Cohesion Evaluation Scale (FACES) IV [12], a self-report assessment developed to capture a family’s balanced and unbalanced levels of cohesion and flexibility. Six scales are included into the FACES IV questionnaire, with medium-large alpha reliability scores (ranging from 0.77 to 0.89) [12]. Here we used the internal cohesion and flexibility scores.

### Data analyses

Raw responses obtained from all participants were extracted from REDCap in a unique file. Data analyses were performed with IBM SPSS Statistics for Windows, version 26 (IBM Corp., Armonk, N.Y., USA).

Frequencies and descriptive statistics were calculated. Raw responses relating to each FED symptom were transformed into a change score (∆) with a no-change value of 0 and ranging from − 50 (extreme worsening) to 50 (extreme improvement), in order to assess change across symptoms.

For the purpose of identifying global improvement or worsening of FED symptoms, for each participant a summary score was calculated by averaging all the endorsed symptoms’ ∆. Normality of this score was tested using the Kolmogorov–Smirnov and Shapiro–Wilk tests.

To obtain a dichotomous value indicating improvement/no change vs. worsening, the summary score was coded as 1 (improvement or no change in FED symptoms for mean values ≥ 0) and 0 (worsening of FED symptoms for mean values < 0).

In order to assess the association between individual and baseline clinical variables on our dichotomous index of symptom change, we performed logistic regressions using the latter as the dependent variable, and state and trait anxiety scores, cohesion and flexibility scores as independent variables.

The relationship between FED diagnosis, age range, COVID-19 infection, self-reported anxiety, home/schooling arrangements, and meal organization and symptoms change was investigated using cross-tabulations (due to low cell numbers in some instances).

## Results

### Sample

Overall, from September 2020 to July 2021 61 patients and their parents were contacted to complete the survey. When the survey was first sent, 20 patients were already in treatment.

A total of 85 raw responses, inclusive of both children and parents’ individual answers, were received, for 61 individual subjects.

Amongst children’s responses, 17 were excluded: 10 were empty, 4 were incomplete, 3 entered an incorrect participation code. Amongst parents’ responses, 10 were excluded because empty. In order to minimize data loss, we decided to keep and analyse participants who had at least 1 questionnaire complete (i.e., either parental or child report) Overall 20 (44.4%) questionnaires were completed by the adolescent only, 13 (28.9%) by parent only, and 12 (26.7%) by both parent and child.

The final sample for analyses consisted of 45 individuals: 82.2% of the participants were female, 75.6% were aged between 14 and 18 years and the most prevalent FED diagnosis among the sample was Anorexia Nervosa (AN) (64.4%). The main characteristics of our sample are shown in Table 1.

**Table 1:** Sample characteristics

	*n*	%
*Gender*		
Female	37	82.2
Male	8	17.8
*Age*		
11–13	11	24.4
14–18	34	75.6
*Language*		
ENG	12	26.7
FR	33	73.3
*FED diagnosis*		
AN	29	64.4
ARFID	3	6.7
BED	3	6.7
BN	2	4.4
OSFED	8	17.8
*Treatment start (pre or post first Lockdown [2020])*		
Before	17	37.8
After	28	62.2

Clinical questionnaires	*n*	Mean (SD)
*STAI-Y*		
State	41	47.29 (14.76)
Trait	41	45.46 (13.13)
*FACES-IV*		
Cohesion dimension score (CDS)	42	2.05 (0.70)
Flexibility dimension score (FDS)	42	1.58 (0.41)

### FED symptoms change during lockdown

**Fig. 2:**
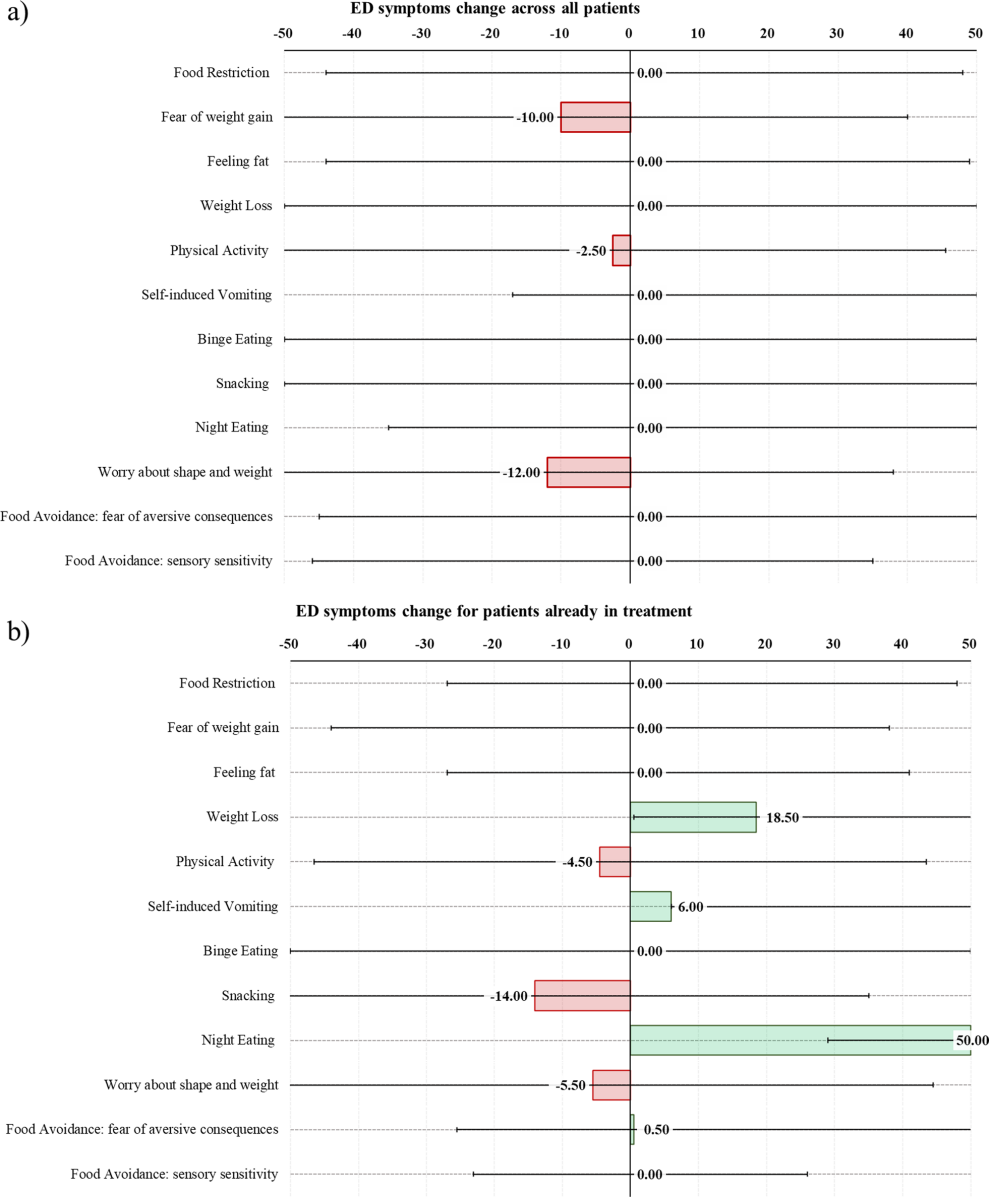
Bar graphs showing FED symptoms change (Median & Ranges) across **a** all patients in our sample and **b** patients who were already in treatment before lockdown. Since data were not normally distributed, median and ranges are presented. The 0 value corresponds to no change, − 50 to extreme worsening and 50 to extreme improvement

Figure 2 shows changes across each symptom investigated.

Overall, when we averaged data across the whole sample, self-induced vomiting, night eating, and feeling fat showed a decrease in frequency/intensity during lockdown as compared to pre-lockdown, whilst fear of weight gain, physical activity, worry about shape and weight showed a worsening (Fig. 2a).

No statistically significant differences emerged when comparing symptom change in subjects already in treatment during lockdown vs. those who were not (all *p* > 0.05). However, at a qualitative level we observed that patients who were already in treatment showed a more pronounced improvement related to weight loss, night eating, and self-induced vomiting (Fig. 2b). In contrast worry about shape and weight, physical activity, snacking, and food avoidance based on sensory sensitivity worsened in those already in treatment vs. those who were not in treatment.

When we investigated change in overall symptomatology 53.3% of individuals (n = 24) showed a worsening in symptomatology, whereas 46.7% (n = 21) reported improvements or no change.

### Correlates of symptom change

Trait and state anxiety, as well as family levels of cohesion and flexibility at evaluation, were not associated with worsening or improvement in symptomatology (all logistic regressions ps > 0.05).

**Fig. 3:**
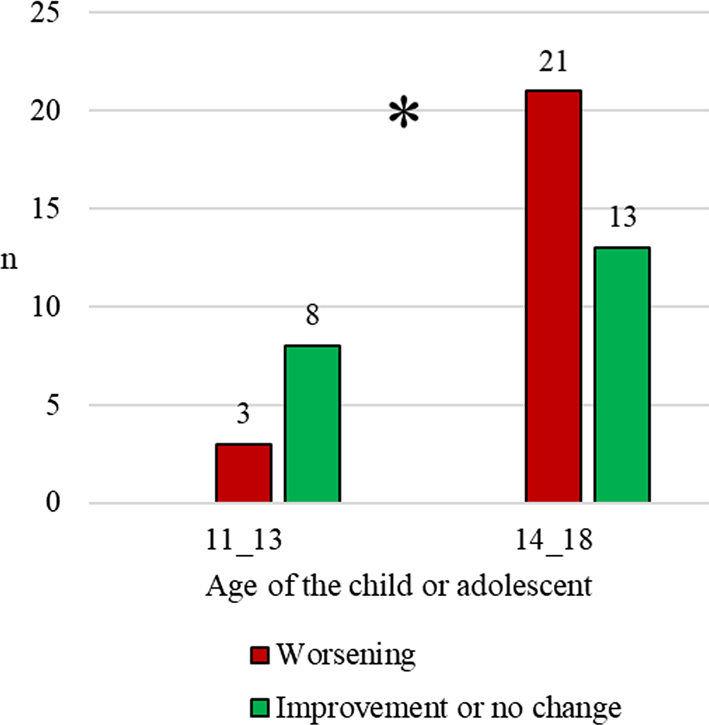
FED symptomatology changes across age ranges (Preadolescents (11–13) vs Teenagers (14–18)). *X^2^ = 3.97, *p* = 0.046

Conversely, age was associated with symptom change during lockdown (Fig. 3): younger patients (preadolescents of 11–13 years of age) were more likely to show improvement of symptoms compared to older patients (teenagers of 14–18 years of age) (X^2^ = 3.97, *p* = 0.046). This was also true when excluding individuals who were newly assessed during the pandemic.

No statistically significant associations emerged between symptom change and individual, family, COVID, and lockdown related variables (i.e., COVID-19 infection in the nuclear family, worry about one’s own or relatives’ health, home and meal arrangements) (see Additional file 1: Table S1). Percentage-wise all individuals who reported improvement or no change in symptoms reported having had all or the majority of their daily meals prepared by a parent and having eaten all or the majority of their daily meals with one or both parents.

In terms of diagnostic categories, those with AN reported the highest level of worsening (58.6%).

However, this varied according to whether they were already in treatment before Spring 2020 (n = 14), or not (n = 15): improvement of symptoms was more common (57.1%) amongst patients with AN who were already in care in our clinic, as expected. Individuals with ARFID (Avoidant Restrictive Food Intake Disorder) were those that reported the highest percentage of no change/improvement (although this group was extremely small (n = 3)).

The impact of COVID-19 lockdown on each diagnostic subgroup is shown in Table 2.

**Table 2:** Impact of COVID-19 lockdown on each FED diagnostic subgroup (Row %)

Feeding and eating disorder diagnosis	Worsening *n* (%)	Improvement or no change *n* (%)
AN (n = 29)	17 (58.6)	12 (41.4)
ARFID (n = 3)	0 (0)	3 (100)
BED (n = 3)	1 (33.3)	2 (66.7)
BN (n = 2)	1 (50)	1 (50)
OSFED (n = 8)	5 (62.5)	3 (37.5)

Twenty-eight (62.2%) subjects who took part in the study were newly evaluated in our specialist unit after the first lockdown in Spring 2020. Amongst these, for 17 (60.7%) the first COVID-19 lockdown was described as the precipitating factor at evaluation.

The vast majority (93.8%, n = 15) of patients who were already in treatment before the first lockdown (n = 17) evaluated access and availability of care during lockdown as satisfactory (despite changes imposed by governmental restrictions).

## Discussion

The aims of the present study were to assess the impact of COVID-19 lockdown on FED symptoms, and to evaluate the potential effect of individual factors, like anxiety and family-related features on symptomatology worsening or improvement in a clinical population of children and adolescents presenting with FED.

Older adolescents appeared more susceptible to symptom worsening during lockdown compared to younger patients. This age-related finding is in line with the effect of age (adults vs. adolescents) found by the study of Schlegl et al. [15] in a clinical, mixed-age population with AN. During the COVID-19 lockdown, adults with AN were in general more affected and reported a greater impairment than adolescents with AN, who in turn reported encouraging and positive eating-related behaviors like being more flexible regarding meals and foods, trying out therapy content, taking on responsibility to recover and working on accepting uncertainty in life [15].

Overall a roughly equal number of patients with FED showed improvement/no change (46.7%) or worsening (53.3%) of symptomatology. This finding is consistent with the available literature in adult [16, 21] and adolescent [2] FED clinical populations. Amongst adults with FED both a deterioration of FED symptomatology during lockdown, especially for those with AN, and also a favorable, beneficial effect on intrafamilial relationships and motivation to recover/comply with treatment were highlighted [15, 16, 21]. Akgül et al. [2] evidenced that the 42.1% of their FED adolescent sample (n = 38) reported feeling an improvement in symptomatology, consistent with our results.

Amongst patients who were first evaluated post lockdown, for 60.7% lockdown was recognized as a precipitating factor for the disorder. COVID-19 lockdown seemed to have a positive effect for youth who were already in treatment in our clinic before Spring 2020.

To date, only few studies investigating how young, clinical populations with FED reacted to and coped with COVID-19 lockdown are available [2, 8, 18, 22]. Moreover, only three other COVID-19-focused investigations, comparable in terms of sample and methodology with our study, have been carried out in adolescents with FED [2, 18, 22]. Spettigue et al. [18] showed that for 40% of their sample of adolescents (n = 48) the COVID-19 pandemic was a trigger for an ED, whereas Vitagliano et al. [22] demonstrated a negative impact of the COVID-19 pandemic on FED symptoms in patients who reported concerns for a negative environmental change during lockdown (63%).

The strength of our study is above all the inclusion of a clinical sample composed by a young population with a broad spectrum of FED, including ARFID. However, our findings have to be understood in light of some limitations. First, a large majority of our sample had AN (64.4%), and the sample size across the other diagnostic categories was small, making a comparison across diagnosis difficult. However, this reflects the diagnostic split of our patient population. Second, data about the impact of lockdown were collected retrospectively. This was due to the time lapse between the start of the pandemic, ethical approval of the study, and study set up. Third, our survey was not validated, however no similar surveys were available when the study begun, and it would have impossible to validate it prior to the beginning of the study. However, we based the survey on available symptom checklists. Lastly, sample size was limited. Additionally, in order to include as many individuals as possible, we relied preferentially on child/adolescent but when this was missing parental report was used as a proxy measure.

## Conclusions

In conclusion, the first COVID-19 lockdown was a ‘mixed bag’ for this clinical sample of children and adolescents with FED. For half of these youth it had a positive effect on FED symptomatology, for others it meant a new disorder onset or the worsening of pre-existing symptoms. Patients with AN reported the highest level of symptomatology worsening, and younger patients showed a greater improvement of symptoms compared to older ones. In contrast to our hypothesis, none of our measured variables (anxiety and family functioning, COVID-19 infection, home and schooling arrangements and meals organization) were associated with worsening or improvement in FED symptomatology. In spite of restrictions, treatment and care provided by our team during lockdown was evaluated as satisfactory by nearly all patients.

Overall our findings highlight that although the pandemic and the related lockdown was a precipitating factor for a FED for many new cases, it had a positive impact on youth who were in treatment. Although we were not able to draw firm conclusions due to the small sample size, there was an indication that the increased meal support might have contributed to an improvement in symptoms for those who were already receiving therapy.

## Supplementary Information

**Additional file 1: Table S1.** Associations between COVID-19 lockdown and related variables and symptom change.

## Data Availability

The datasets generated during and/or analyzed during the current study are available from the corresponding author on reasonable request.

## Abbreviations

AN: Anorexia nervosa
ARFID: Avoidant restrictive food intake disorder
FED: Feeding and eating disorders
FACES: Family adaptability and cohesion evaluation scale
ED: Eating disorders
REDCap: Research electronic data capture
STAI: State-trait anxiety inventory
STAIC: State-trait anxiety inventory for children
