# Supplementary material for: Locked down with my eating disorder: a retrospective study on the impact of COVID-19 lockdown on adolescents with eating disorders
Source: J Eat Disord. 2023 May 10;11:71. doi: 10.1186/s40337-023-00792-1 (PMC10171150; doi:10.1186/s40337-023-00792-1)
Supplement: Supplementary file 1 — Additional file 1: Table S1. Associations between COVID-19 lockdown and related variables and symptom change. [file 40337_2023_792_MOESM1_ESM.docx]

**SUPPLEMENTARY**

**Table 1SM.** Associations between COVID-19 lockdown and related variables and symptom change (Column %).

|  | | **Worsening**  ***n* (%)** | **Improvement or No change**  ***n* (%)** | **Fisher’s Exact Test**  **p value** |
| --- | --- | --- | --- | --- |
| **COVID-19 in the family**  **(n = 43)** | Yes | 5 (22.7%) | 4 (19.0%) | p = 1.000 |
|  | No | 17 (73.3%) | 17 (81.0%) |  |
| **Anxious about one’s health**  **(n = 44)** | Yes | 10 (43.5%) | 6 (28.6%) | p = 0.360 |
|  | No | 13 (56.5%) | 15 (71.4%) |  |
| **Anxious about family member’s health**  **(n = 44)** | Yes | 11 (47.8%) | 10 (47.6%) | p = 1.000 |
|  | No | 12 (52.2%) | 11 (52.4%) |  |
| **Parents working from home**  **(n = 45)** | Both | 12 (50.0%) | 7 (33.3%) | p = 0.480 |
|  | One | 8 (33.3%) | 11 (52.4%) |  |
|  | None | 4 (16.7%) | 3 (14.3%) |  |
| **Meals eaten with parents**  **(n = 40)** | All or most with at least one parent | 22 (95.7%) | 17 (100%) | p = 0.153 |
|  | None | 1 (4.3%) | 0 |  |
| **Meals prepared by parents**  **(n = 40)** | All or most by at least one parent | 21 (91.3%) | 17 (100%) | p = 0.085 |
|  | None | 2 (8.7%) | 0 |  |
